# High-performance grating couplers on 220-nm thick silicon by inverse design for perfectly vertical coupling

**DOI:** 10.1038/s41598-023-45168-2

**Published:** 2023-10-23

**Authors:** Mingxiang Yang, Yunjie Yan, Zhenlin Wu, YiYing Gu, Shiyuan Zhao, Geert Morthier, Mingshan Zhao

**Affiliations:** 1 Photonics Research Center, School of Optoelectronic Engineering and Instrumentation Science, Dalian University of Technology, Dalian, 116024 China; 2 Department of Information Technology, Ghent University-IMEC, 9052 Ghent, Belgium

**Keywords:** Photonic devices, Silicon photonics

## Abstract

Efficient grating couplers (GCs) for perfectly vertical coupling are difficult to realize due to the second-order back reflection. In this study, apodized GCs (AGCs) are presented for achieving perfectly-vertical coupling to 220 nm thick silicon-on-insulator (SOI) waveguides in the C-band. We compare the performance of the AGCs to that of uniform GCs (UGCs) and demonstrate the superiority of the former. The AGCs were obtained through inverse design using gradient-based optimization and were found to effectively suppress back reflection and exhibit better matching to the Gaussian beam profile. The design and measurement results show that AGCs have a 3 dB lower coupling loss than UGCs. We fabricated focusing AGCs by electron beam lithography with a single, 70 nm shallow etch and a minimum feature size of 100 nm, which makes them compatible with CMOS technology. The AGCs achieved a coupling efficiency of −5.86 dB for perfectly vertical coupling. Overall, our results demonstrate the potential of AGCs for achieving high-performance coupling in the C-band on the SOI platform.

## Introduction

Silicon photonics has a high potential as an integrated photonics platform because of its scalability. It has already been efficiently used for the photonics computing, the data-center interconnect, and the optical telecommunications markets^1^. Recently, there has been a growing interest in using silicon photonics for free-space optical (FSO) communication due to its potential for chip-scale miniaturization and reduction in cost, size, weight, and power (CSWaP)^2,3^. The optical coupling between silicon chips and free space is key to achieving chip-scale systems. Up till now, GCs have been primarily used for fiber coupling^4,5^. Due to the three-dimensional (3D) character of FSO, grating GCs offer much more flexibility in terms of arbitrary coupling position on the chip and are therefore more suitable for the optical coupling of free-space beams. The free-space optical beams should be collimated and the optical mode field should have a Gaussian-like distribution in 3D FSO systems^6^. Especially in some focal plane array (FPA) systems, GCs can be used as receiver to receive 3D FSO information, making the system miniaturized and flat, as show in the supplement materials of reference^7^ and the direction of the free-space beams should be perfectly vertical to the surface of the silicon chips^8–10^.In addition, the perfect vertical optical coupling makes the alignment for wafer-level testing and for coupling of light to multicore fibers easier^11,12^. Therefore, high-performance GCs for perfectly vertical coupling are extremely important elements in silicon photonics. The primary challenge is the severe back reflection problem caused by perfectly vertical coupling resulting from second-order Bragg reflections^13–16^.

In recent years, significant effort has been dedicated to designing and optimizing perfectly vertical GCs. Various structures such as an asymmetric grating structure^13^, a grating on a tilted silicon membrane^17^, subwavelength metamaterials, and a dual-layer grating coupler^18^ have been developed to enhance the directivity of these GCs and reduce back reflection. However, these structures require complex fabrication techniques that are not compatible with standard silicon photonics processes. Fortunately, the emerging inverse design approach offers a novel method for designing GCs, providing more degrees of freedom to design AGCs that match the Gaussian beam, resulting in better coupling efficiency^19^. HP Labs have reported experimental coupling losses of 2 dB for inverse-designed GCs with a partial etch depth of 159 nm and a Si height of 304 nm^20^. However, these dimensions are not compatible with commonly-used silicon photonics processes that rely on a 220 nm thick silicon device layer.

This work presents novel, compact, AGCs by inverse design for perfectly vertical coupling between free-space and an optical waveguide, designed and fabricated with a simple 70 nm shallow etch available from most silicon photonics foundries. The optimized and experimentally demonstrated coupling efficiency of AGCs are −3.0 dB and −5.86 dB respectively in the C-band. The AGCs obtained by inverse design using gradient-based optimization are shown to reach a low coupling loss that has improved by 3 dB over UGCs. Table 1 presents a comparison of the design and measurement results with those obtained in previous reports. Our results are the best performance of focusing perfectly vertical GCs in the C-band on 220 nm silicon-on-insulator with a single 70 nm etch, compatible with CMOS technology.

**Table 1:** Comparison to literature of the perfectly vertical GCs.

θ	λ (nm)	CE_Sim_ (dB)	CE_Mes_ (dB)	Si (nm)	Description	References	Year
0°	1550	− 1.0	–	220	Poly-silicon	^13^	2007
0°	1550	− 1.6	–	300	Dual-layer	^18^	2015
0°	1550	− 0.9	−1.5	220	2-step etching	^15^	2017
0°	1310	− 1.8	−2.4	220	tilted silicon membrane	^17^	2017
0°	1550	− 2.0	−2.7	380	Poly-silicon	^12^	2018
0°	1310	− 0.3	−1.8	304	159 nm etching	^20^	2021
0°	1550	− 3.0	−5.9	220	70 nm etching	*	2023

*:This work.

## Result and discussion

### The problems of UGCs

**Figure 1:**
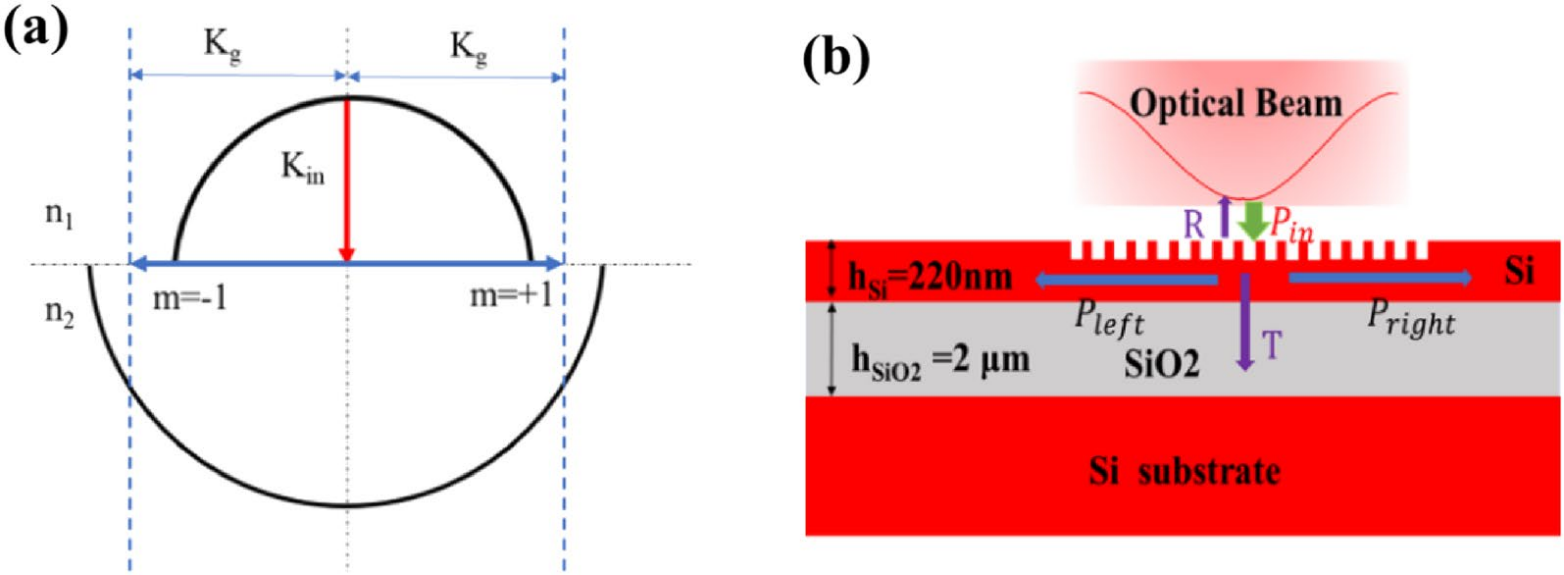
(**a**) Wave-vector diagram of the perfectly vertical uniform GC for input coupling. (**b**) Cross-section view of the uniform perfectly vertical GCs.

**Figure 2:**
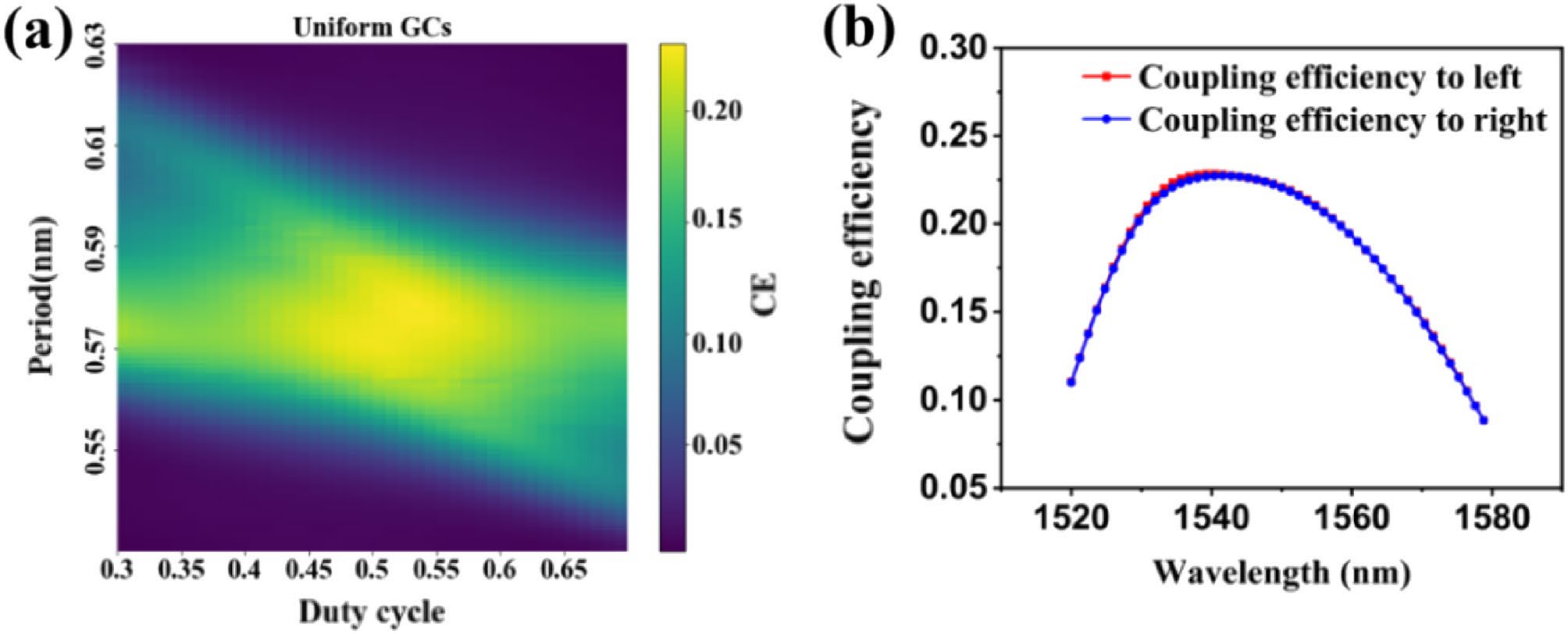
(**a**) The coupling efficiency (CE) of UGCs at a wavelength of 1550 nm was obtained from 2D-FDTD simulation. (**b**) The coupling efficiency of left and right of UGCs for a duty cycle of 0.5 and period of 585 nm.

The design of GCs is based on the fundamental principle of satisfying the Bragg diffraction condition. In the case of uniform, single-etch, perfectly vertical GCs shown in Fig. 1a, the incident optical beam has an angle of 0°. Due to the symmetry, the leftward and rightward propagating guided modes will meet the Bragg condition at the same wavelength for diffraction order $m=-1$ and $m=+1$. The cross-section and optical power transmission of perfectly vertical UGCs are depicted in Fig. 1b. To design UGCs, we utilized a typical silicon-on-insulator (SOI) structure consisting of a 220 nm silicon device layer and a 2 μm buried oxide layer on a silicon substrate. The uniformly etched grooves induce effective index modulation, resulting in the diffraction of the incident optical beam in both leftward and rightward directions. The UGCs were simulated using the 2D finite-difference time-domain (FDTD) method provided by Lumerical Inc^21^. We performed a parameter optimization by defining a 70 nm shallow etch and sweeping the period and duty cycle of the GC. The optimal parameters, as shown in Fig. 2a, were found to be a period of 585 nm and a duty cycle of 0.50 at a wavelength of 1550 nm. The corresponding coupling efficiency was determined to be 23%. Due to the geometric symmetry, almost an equal amount of optical power is coupled to both the left and right sides, as illustrated in Fig. 2b.

The coupling efficiency of UGCs is primarily limited by two factors. Firstly, the symmetry of the grating structure causes the coupled optical power to be evenly diffracted towards both the left and right sides. Secondly, there is a mismatch between the waveguide grating mode and the free space optical beam field. This occurs because the period and duty cycle of the uniform grating are fixed, resulting in a fixed leakage factor, and leading to a diffraction pattern that doesn’t match the Gaussian distribution of the free-space optical beam. The apodized grating coupler is the key to improving the coupling efficiency by breaking the symmetry and changing the leakage factor of the grating structure to match the Gaussian beam profile.

### Design and optimization of the perfectly vertical GCs

The AGCs are designed using the gradient-based inverse design methods ^22^ that are included in the Python suite called LumOpt. Compared to the previous particle swarm optimization (PSO), relying on random perturbations, the gradient-based optimization algorithm enables obtaining the best solution in fewer iterations. Moreover, gradient-based inverse design methods can realize super multi-parameter optimization through the adjoint method in electromagnetic design problems. For the design of GCs, the number of degrees of freedom for optimization depends on the number of periods of the grating. Because of this, we can design the apodized grating with different leakage factors for every period to achieve the Gaussian distribution of the field diffracted by the grating. Thus, the power diffracted by the grating P(x)^16,23,24^ can be expressed as:

1 $$\begin{array}{c}P\left(x\right)=P\left(0\right)exp\left[-2\eta x\right]\end{array}$$

2 $$ \begin{array}{*{20}c}    {\eta \left( x \right) = \frac{{G^{2} \left( x \right)}}{{2 \times \left[ {1 - \mathop \smallint \nolimits_{0}^{x} G^{2} \left( t \right)dt} \right]}} = \frac{{e^{{{\raise0.7ex\hbox{${ - x^{2} }$} \!\mathord{\left/ {\vphantom {{ - x^{2} } 2}}\right.\kern-\nulldelimiterspace} \!\lower0.7ex\hbox{$2$}}}} }}{{2 \times \left[ {1 - \mathop \smallint \nolimits_{0}^{x} G^{2} \left( t \right)dt} \right]}}}  \\   \end{array}  $$

where $x$ is the position of the grating, $\upeta \left(\mathrm{x}\right)$ is the variable leakage factor along the grating by varying the widths of rib and groove for every period, G($x$) is the Gaussian mode field.

The same SOI stack is also used to design AGCs with 70 nm shallow etching. The Gaussian optical beam at 1550 nm wavelength with an effective mode diameter of 10.4 μm (waist diameter) is used as the input beam during the design. The angle between the input beam and the grating surface is set to 90°, ensuring a perfectly vertical orientation. The objective of the optimization process is to maximize the mode overlap between the scattered fields from the grating and the predefined field profile. In order to achieve this, the intensity of the light diffraction from the grating should exhibit a gradual increase and decrease along the direction of the grating to match the Gaussian input beam. Consequently, the leakage factor of the grating should also gradually grow and then diminish in the same direction. To fulfill this requirement, an apodized grating structure is employed, which can accommodate this non-linear variation. To rapidly generate electromagnetic structures with numerous degrees of freedom using 2D-FDTD simulation, a gradient-based optimization technique is adopted, leveraging the adjoint method. This approach facilitates the efficient design of electromagnetic structures. Simultaneously, a minimum feature size is enforced to ensure the manufacturability of the final design.

**Figure 3:**
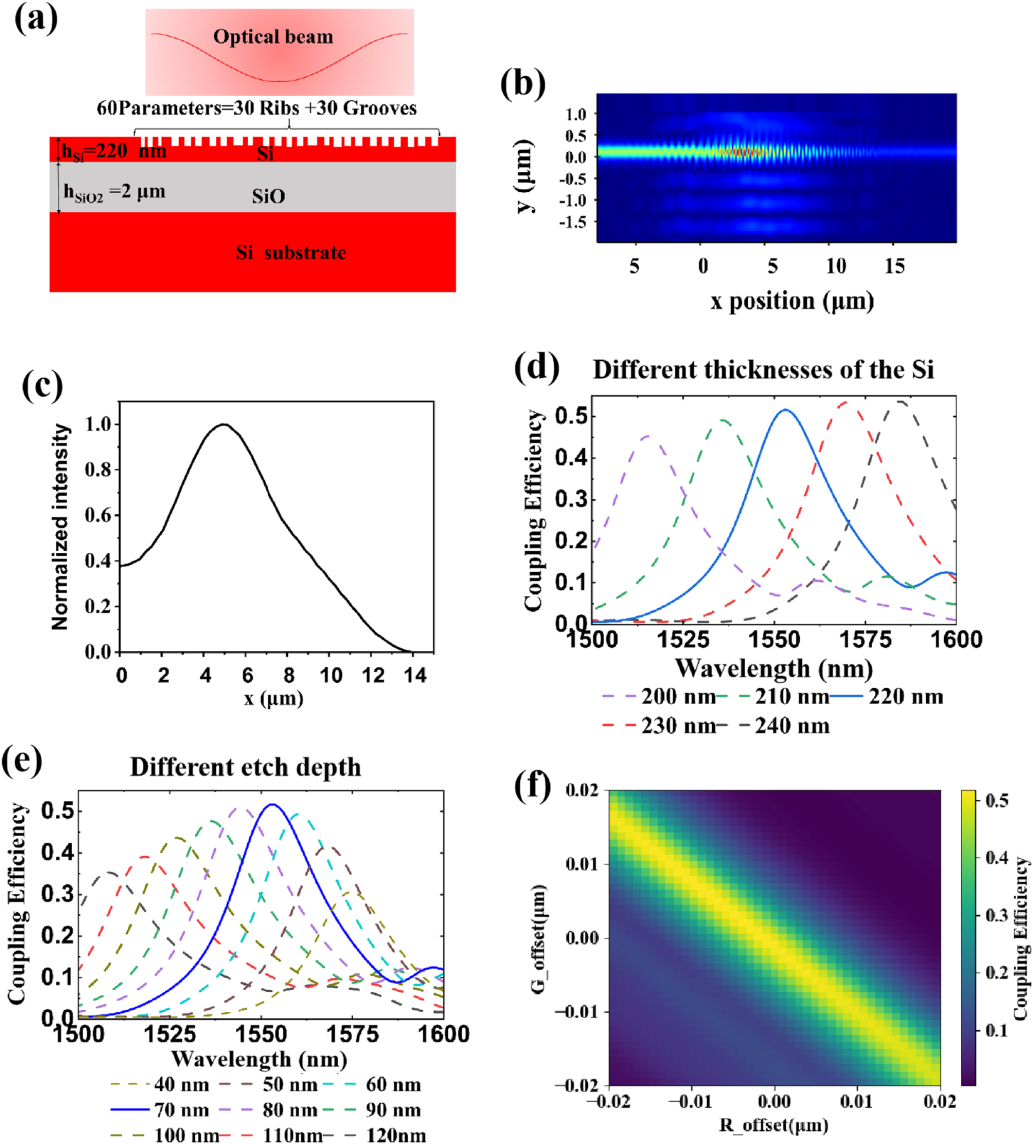
(**a**) The schematic diagram of the geometry of the AGCs. (**b**) The simulated electric field of the AGCs. (**c**) The intensity of the light distribution along the direction of the grating. (**d**) The simulation results of coupling efficiency with different thicknesses of the silicon layer for a 70 nm etch depth. (**e**) The coupling efficiency of GCs with different etch depths for a 220 nm thick silicon. (**f**) The sensitivity of coupling efficiency with variations in rib and groove widths.

**Figure 4:**
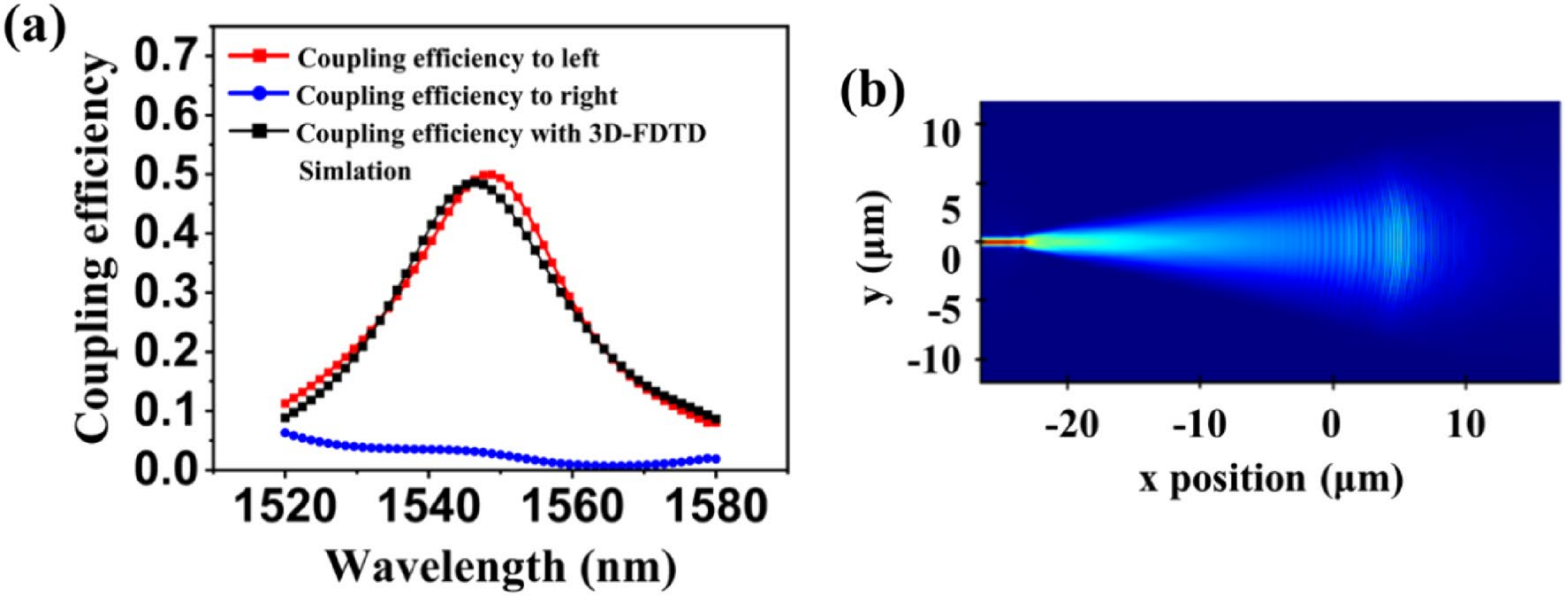
(**a**) The coupling efficiency of the AGCs. The red and blue lines are the results of simulation with 2D-FDTD. The black line is the results of simulation with 3D-FDTD for the focusing AGCs. (**b**) Electric intensity distributions of apodized grating coupling from 3D-FDTD simulation.

The schematic diagram of the geometry of the optimized AGCs is illustrated in Fig. 3a. The design consists of 30 periods (P), with rib (R) and groove (G) dimensions specified in Table 2. During the optimization process, the minimum width and spacing are constrained by a feature size of 100 nm to simplify fabrication. The varying widths of the rib and groove for each period introduce asymmetry to the grating geometry. Figure 3b presents the simulated electric field of the optimized AGCs. It is visually evident that the field profile of the apodized grating gradually weakens from the center towards the two sides. In Fig. 3c, the normalized intensity of the optical diffraction from the apodized grating exhibits a Gaussian-like distribution, which aligns more closely with the input Gaussian beam, resulting in enhanced coupling efficiency. The distribution of optical power confirms the superior performance of the AGC obtained through inverse design. In Fig. 4a, it is observed that the majority of the optical power is vertically coupled to the left side, while the power on the right side is comparatively weaker. This indicates a high level of directivity, with the AGC achieving over 50% (3.0 dB) coupling efficiency based on the 2D-FDTD simulation.

**Table 2:** The comparison of the geometry of the AGCs with the design and measurement.

	P	1	2	3	4	5	6	7	S	9	10	11	12	13	14	15
Design	*Rl* (nin)	–	454	4S9	564	SSI	476	361	46S	551	401	397	361	3^4	396	405
Measurement	R; < nm)	–	445	435	435	305	425	305	395	465	335	325	2S5	325	335	345
Discrepancy	AR(nm)	–	9	54	79	76	51	56	73	S6	66	72	76	49	61	60
Design	G,(nm)	100	100	100	100	109	145	117	124	157	173	190	1S6	187	155	163
Measurement	Gj(nm)	150	130	160	140	160	190	ISO	190	210	210	240	240	260	210	210
Discrepancy	iGfnm)	−50	−30	−60	−40	−51	−45	−63	−64	−53	−37	−50	−54	−73	−55	−42

	P	16	17	IS	19	20	21	22	23	24	25	26	27	2S	29	30
Design	Rl (nin)	262	233	102	439	395	424	1S9	417	125	427	37S	140	3S5	133	351
Measurement	R; < nm)	225	1S5	100	375	335	375	135	335	100	375	325	115	325	115	295
Discrepancy-	AR(nm)	37	4S	2	64	60	49	54	S2	25	52	53	25	60	23	56
Design	G^nin)	2S6	100	173	131	159	136	150	104	140	1S4	144	192	116	220	171
Measurement	Gjfnin)	350	140	240	130	210	ISO	190	160	ISO	230	190	210	120	260	230
Discrepancy	AG(nm)	−64	−40	−67	−49	−51	−44	−40	−56	−40	−46	−56	−IS	−4	−40	−59

The top silicon layer of a standard SOI wafer typically has a thickness of 220 nm. However, it was found that the SOI used for the fabrication had a 10 nm thicker silicon layer. The coupling efficiency dependence on the thickness is presented in Fig. 3d. The increase in the effective index of the gratings with thicker silicon causes the peak wavelength to shift to longer wavelengths. In addition, the etching depth also affects the performance of GCs. Figure 3e shows the coupling efficiency with different etching depths, among which 70 nm is the best etching depth. Furthermore, Fig. 3f displays the sensitivity of coupling efficiency to variations in rib and groove widths. At the peak of coupling efficiency, the allowable tolerance for both the rib width and groove width is approximately ± 2 nm. Notably, when the period width of the AGCs remains constant, meaning that any increase in rib width is accompanied by a decrease in groove width in equal proportions, the coupling efficiency achieves optimal performance.

To validate the accuracy of the geometric structure, the apodized grating coupler is simulated using a 3D-FDTD simulation environment. Additionally, a focusing grating with circular lines is employed to reduce the footprint, making it more suitable for high-density photonic integrated circuits. The electric intensity distributions of the optical field at 1550 nm are depicted in Fig. 4b, illustrating the efficient coupling of light into the left waveguide through the compact taper. Moreover, the coupling efficiency obtained from the 3D-FDTD simulation, as shown in Fig. 4a, exhibits a high degree of consistency with the results obtained from the 2D-FDTD simulation. This further confirms the accuracy of the geometric structure and the reliability of the simulation approach.

### Fabrication and experiment

**Figure 5:**
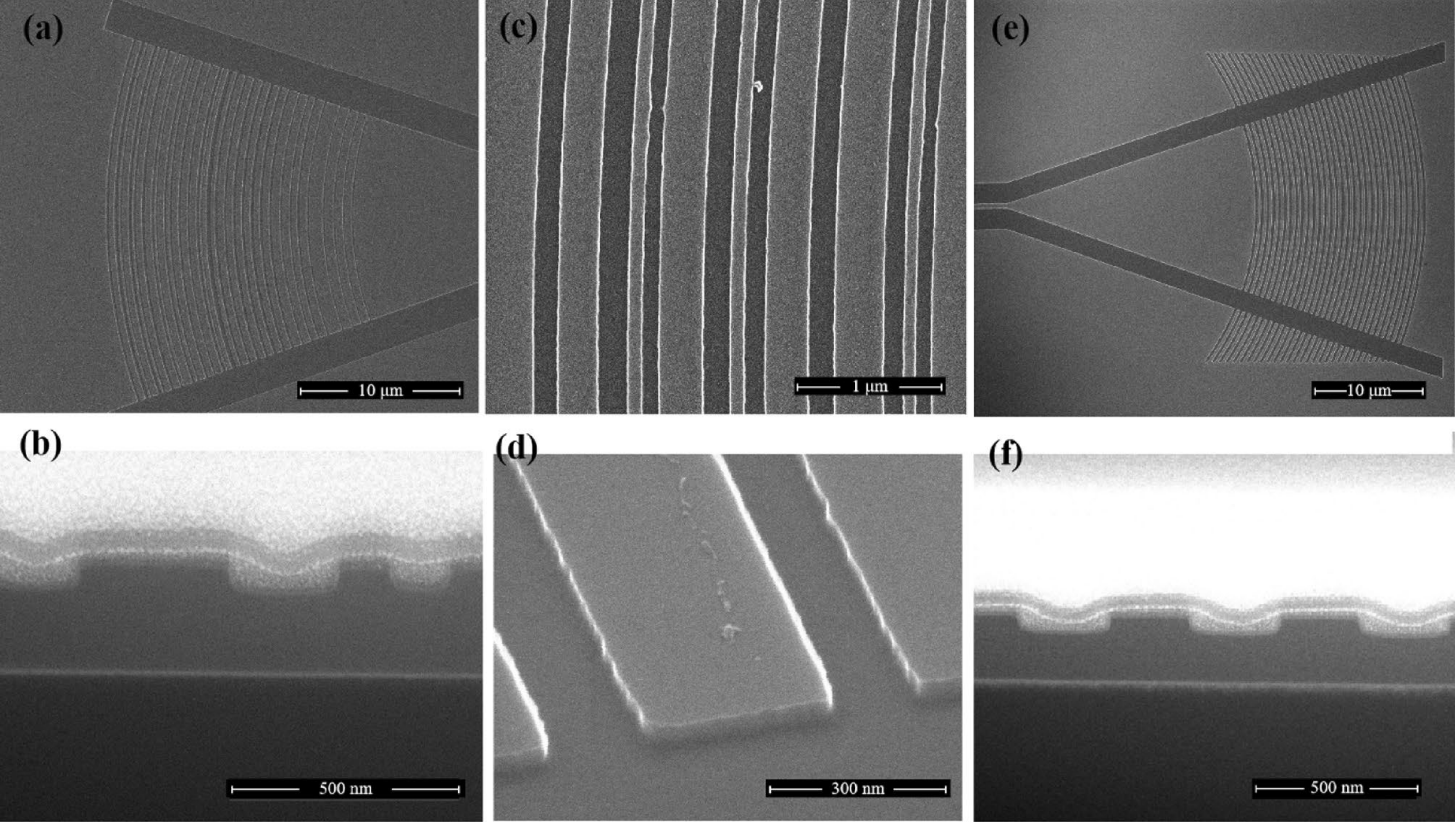
(**a**) SEM top view images of AGCs. (**b**) Cross-section view images of the AGCs. (**c**) Partial SEM top view image of AGCs. (**d**) Detail enlargement SEM view image of AGCs. (**e**) SEM top view images of UGCs. (**f**) Cross-section view images of the UGCs.

On a standard SOI wafer with a silicon layer thickness of 220 nm and a box layer thickness of 2 μm, we fabricated both uniform and AGCs. The GCs and the connecting waveguide were defined using different etching techniques. The GCs were created with 70 nm shallow etching, while the connecting waveguide underwent 220 nm full etching. These fabrication processes involved the use of electron beam lithography (EBL) and reactive ion etching (RIE). The minimum feature size of 100 nm can be fabricated using these processes to attain the minimum feature of the designed AGCs. For measurement purposes, two GCs with the same structure were connected via a straight waveguide that was 500 μm in length. Figure 5 provides scanning electron microscope (SEM) images, showcasing both top views and cross-section views of the fabricated uniform and AGCs. The sidewall roughness is about 5-10 nm visually, as shown in Fig. 5c, and further enlarged in Fig. 5d, which will has an adverse effect on the efficiency of the grating coupler. The uniform grating coupler has a compact footprint of 32 × 42 μm^2^, while the AGC has a slightly smaller footprint of 27 × 40 μm^2^, indicating its compact design.

**Figure 6:**
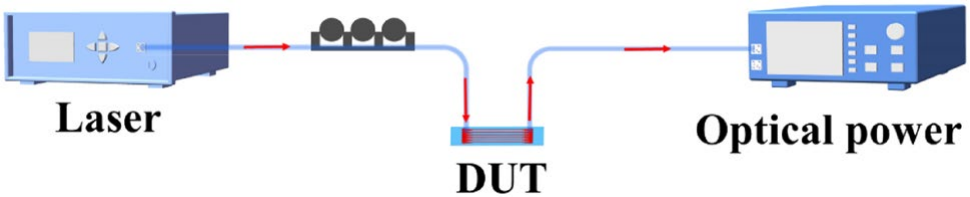
Measurement setup for the perfectly vertical GCs.

For optical characterization, we employed the measurement setup illustrated in Fig. 6. A tunable laser (Santec TSL-550) was utilized to generate light within the wavelength range of 1500 nm to 1630 nm. To achieve TE polarization, a polarization controller was employed to adjust the light accordingly. The optical power emitted by the output grating coupler (GC) was measured using a benchtop optical power meter (Newport 2936-R).To obtain the transmission spectra of the GCs, we synchronized the laser and the optical power meter using Python code. The input and output couplers for 0° were realized by utilizing standard SMF-28 fibers with a Gaussian mode diameter of 10.4 μm (waist diameter). These fibers can be considered free-space Gaussian optical beams. The device under test (DUT) was positioned between the input and output couplers for measurement purposes. In particular, achieving precise vertical coupling is indeed a challenging task. The manual adjustment process introduces inherent errors in the alignment, and any deviation in angle can significantly impact the experimental results.

**Figure 7:**
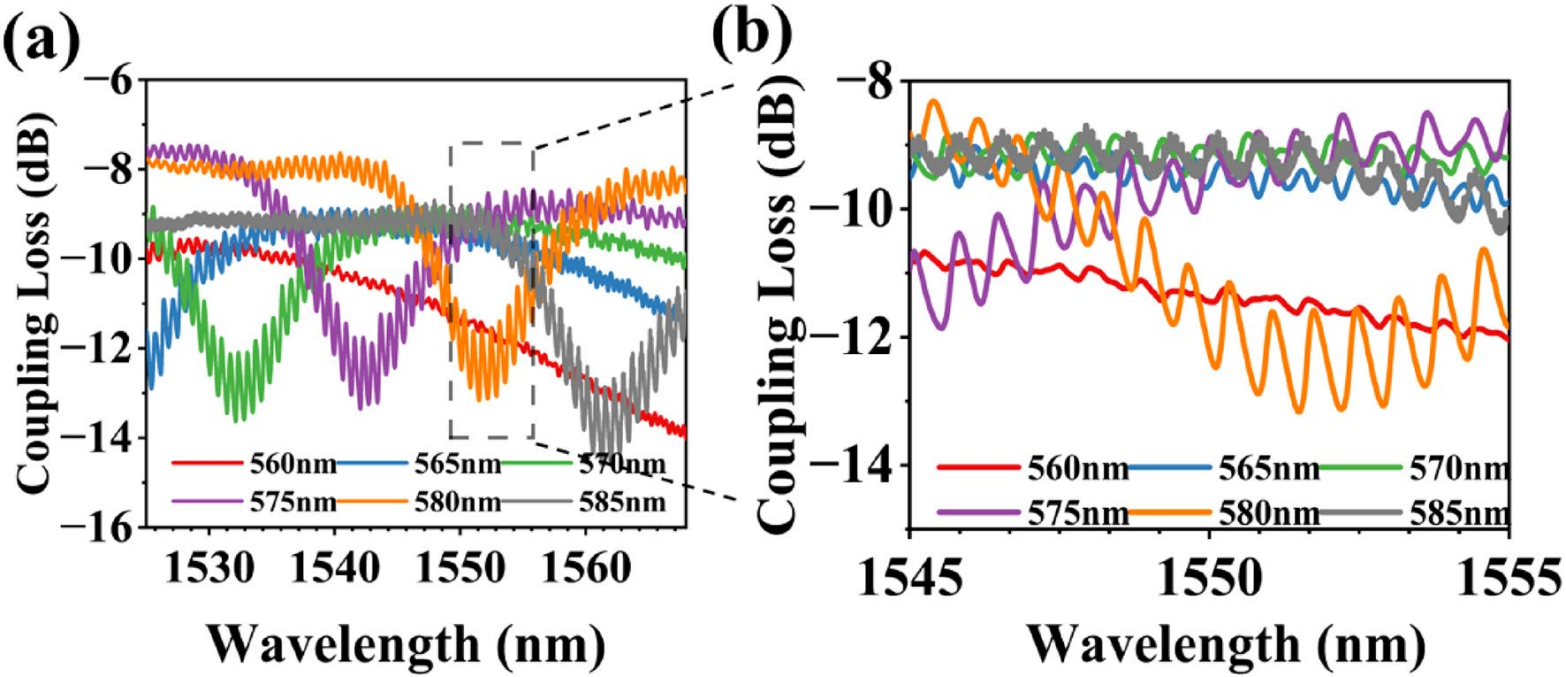
(**a**) Measured results of the UGCs for different periods from 560 to 585 nm. (**b**) Zoom-in measured results.

The measured results of the UGCs are given in Fig. 7a, which shows coupling loss as a function of wavelength The measurements were conducted for different periods ranging from 560 to 580 nm with a step size of 5 nm, while maintaining a constant duty cycle of 0.50. One can find in the zoom-in, in Fig. 7b, that the peak coupling loss of 8.9 dB at 1550 nm is purple and corresponds to a period of 575 nm, which is different from the best design result of 585 nm. This is due to the instability of the manufacturing process. It can be also found that the transmission spectrum has a ripple. According to the resonance formula $\Delta {\lambda }_{FSR}=\frac{{\lambda }^{2}}{{n}_{g}L}$ , the length L of the resonant cavity can be calculated to be approximately equal to the length 500 μm of the waveguide connecting two GCs. This is because the back reflection, caused by the second-order diffraction which also meets the Bragg diffraction condition, creates a resonance effect between the two gratings. This reflection is made worse by the perfectly vertical beam and the lower coupling efficiency.

**Figure 8:**
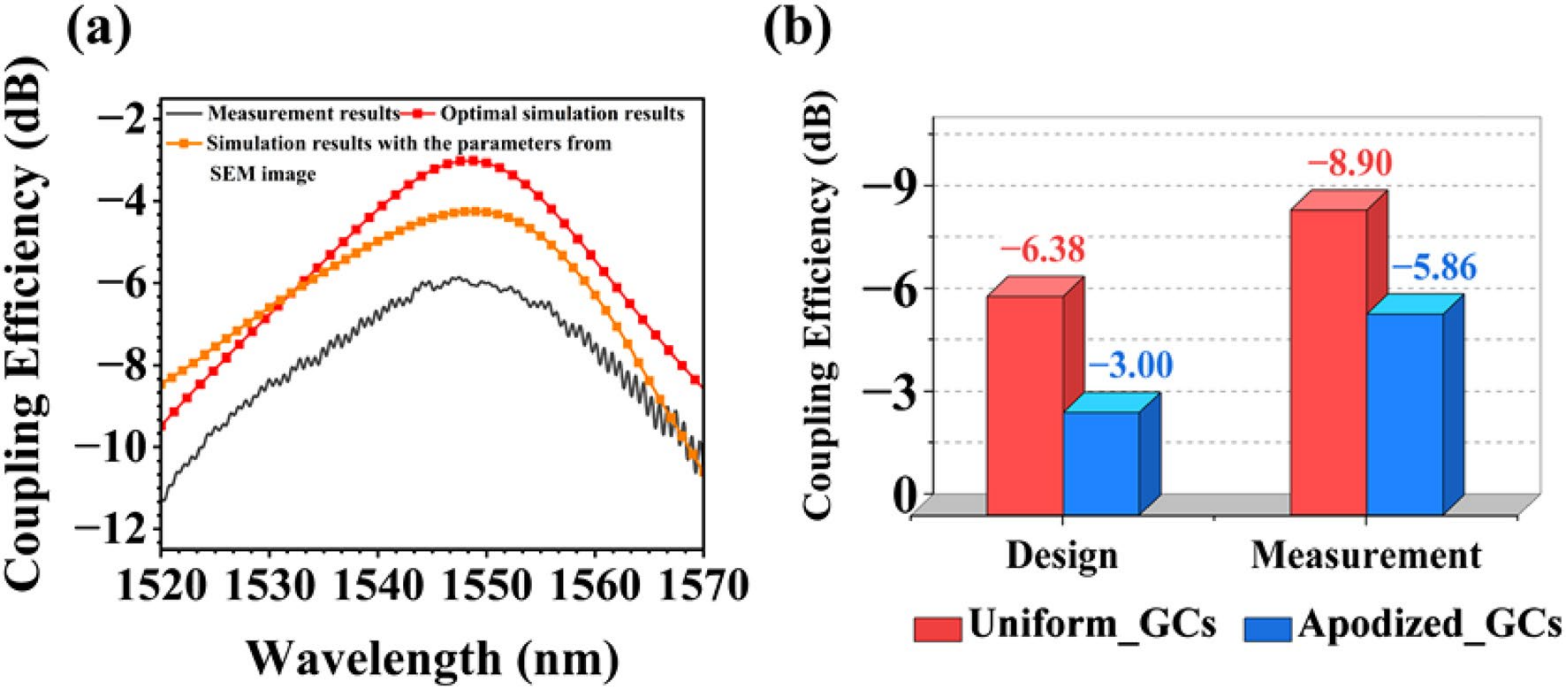
(**a**) The measurement result, optimal simulation result, and the simulation result with parameters from the SEM image of the apodized grating coupler. (**b**) Comparison of design and test results of UGCs and AGCs.

The measured transmission spectrum of the AGCs over a wavelength range (1520 nm ~ 1570 nm) is shown in Fig. 8a. The peak of the coupling loss is 5.86 dB/facet for the TE mode at the wavelength of 1547 nm. It can also be seen from Fig. 8a that the transmission spectrum of the AGC is smoother than that of Fig. 7, which further proves that the back reflection of the AGC is effectively suppressed. In addition to the simulation results for the optimum grating structure, Fig. 8a also contains the simulation results for the grating coupler, as if is fabricated, i.e. for a silicon thickness of 230 nm, an etch depth of 56 nm and for rib and groove widths in Table 2 that were extracted from the SEM image. The simulated coupling efficiency then decreased to −4.25 dB. It’s worth noting that the observed discrepancies between the simulated and measured results are notably larger than the ± 2 nm tolerance indicated in Fig. 3f. However, it's crucial to emphasize that despite these discrepancies in grating dimensions, the coupling efficiency can still achieve optimal performance. We've observed a consistent trend wherein an increase in rib width is accompanied by a decrease in groove width while maintaining a constant period, as corroborated by the data presented in Table 2.

In Fig. 8b, we compare the design and measurement results for both the uniform and AGCs, which demonstrate that the coupling efficiency of AGCs is improved by about 3 dB compared to that of the uniform grating coupler in both cases. This significant enhancement underscores the superior directionality of AGCs. However, it's important to address the observed discrepancy between experimental and simulation results. Even when considering more realistic parameter values for the fabricated grating, the experimental results fall short of the simulated expectations. One key factor contributing to this disparity is the impact of sidewall roughness, as seen in Fig. 5c and magnified in Fig. 5d. Additionally, achieving precise vertical coupling between AGCs and the optical fiber proves to be a formidable challenge, introducing inherent errors in alignment.

## Conclusions

In conclusion, this study addressed the limitations of UGCs for perfectly vertical incidence and proposed the use of efficient AGCs designed through inverse design methods. The optimized AGCs demonstrated optical scattering with a Gaussian-like distribution, resulting in improved coupling efficiency by better matching the input Gaussian beam. Fabrication of both UGCs and AGCs on a standard SOI platform was successfully achieved. The simulated and measured coupling efficiencies of the optimized AGCs were found to be −3.0 dB and −5.86 dB, respectively. This represents a 3 dB improvement compared to UGCs. The compact focusing AGCs had a footprint of 27 × 40 μm^2^, making them suitable for integration in larger arrays. To the best of our knowledge, these experimental results represent the highest achieved coupling efficiency on a 220 nm thick Si platform with a simple 70 nm etch depth and no bottom mirrors. Furthermore, the proposed AGCs are compatible with CMOS technology. The high-performance AGCs for perfectly vertically coupled beams hold significant potential for applications in FPA systems and 3D FSO information processing systems.

## Data Availability

The data that support the findings of this study are available from the corresponding authors upon reasonable request.
